# Information Field Theory and Artificial Intelligence

**DOI:** 10.3390/e24030374

**Published:** 2022-03-07

**Authors:** Torsten Enßlin

**Affiliations:** 1 Max Planck Institute for Astrophysics, Karl-Schwarzschild-Strasse 1, 85748 Garching, Germany; ensslin@mpa-garching.mpg.de; 2 Physics Department, Ludwig-Maximilians-Universität München, Geschwister-Scholl-Platz 1, 80539 Munich, Germany

**Keywords:** information field theory, artificial intelligence, generative models, variational inference

## Abstract

Information field theory (IFT), the information theory for fields, is a mathematical framework for signal reconstruction and non-parametric inverse problems. Artificial intelligence (AI) and machine learning (ML) aim at generating intelligent systems, including such for perception, cognition, and learning. This overlaps with IFT, which is designed to address perception, reasoning, and inference tasks. Here, the relation between concepts and tools in IFT and those in AI and ML research are discussed. In the context of IFT, fields denote physical quantities that change continuously as a function of space (and time) and information theory refers to Bayesian probabilistic logic equipped with the associated entropic information measures. Reconstructing a signal with IFT is a computational problem similar to training a generative neural network (GNN) in ML. In this paper, the process of inference in IFT is reformulated in terms of GNN training. In contrast to classical neural networks, IFT based GNNs can operate without pre-training thanks to incorporating expert knowledge into their architecture. Furthermore, the cross-fertilization of variational inference methods used in IFT and ML are discussed. These discussions suggest that IFT is well suited to address many problems in AI and ML research and application.

## 1. Motivation

Determining the concrete configuration of a field from measurement data is an ill-posed inverse problem, as physical fields have an infinite number of degrees of freedom (DoF), whereas data sets are always finite in size. Thus, the data provide a finite number of constraints for only a subset of the infinitely many DoF of a field. In order to infer a field, the remaining of its DoF need, therefore, to be constrained via prior information. Fortunately, physics provides such prior information on fields. This information might either be precise, like ∇·B=0 in electrodynamics, or more phenomenological, in the sense that a field shaped by a certain process can often be characterized by its *n*-point correlation functions. Having knowledge on such correlations can be sufficient to regularize the otherwise ill-posed field inference problem from finite and noisy data such that meaningful statements about the field can be made.

As a formalism for this, information field theory (IFT) was introduced [1,2] following and extending earlier lines of work [3,4]. IFT is information theory for fields. It investigates the space of possible field configurations and constructs probability densities over those spaces in order to permit Bayesian field inference from data. It has been applied successfully to a number of problems in astrophysics [5,6,7,8,9,10,11,12,13,14,15,16,17,18], particle physics [19,20,21], and elsewhere [22,23,24,25,26]. Here, the relation of IFT with methods and concepts used in artificial intelligence (AI) and machine learning (ML) research are outlined, in particular with generative neural networks (GNNs) and in the usage of variational inference. The presented line of arguments summarizes a number of recent works [27,28,29,30,31,32].

The motivation for this work is twofold. On the one hand, understanding conceptual relations between IFT, ML, and AI techniques allows us to transfer computational methods between these domains and to develop synergistic approaches. This article will discuss such. On the other hand, the current success of deep learning techniques for neural networks has let them appear as a synonym for AI in the public perception. This has consequences for decisions about which kind of technologies get scientific funding. The point this paper is trying to make is that if deep learning qualifies as AI in this respect, then this should also apply to a number of other techniques, including those based on IFT.

The paper is organized as follows. IFT is briefly introduced in Section 2 in its most modern incarnation in terms of standardized, generative models. These are shown to be structurally similar to GNNs in Section 3. The structural similarity of IFT inference and GNN training problems allows for a common set of variational inference methods, as discussed in Section 4. Section 5 concludes on the relation of IFT methods and those used in AI and ML and gives an outlook on future synergies.

## 2. Information Field Theory

### 2.1. Basics

IFT allows us to deduce fields from data in a probabilistic way. In order to be able to apply probability theory onto the space of field configurations, a measure in this space is needed. Although no canonical mathematical measure on function spaces exists, for IFT applications, the usage of Gaussian process measures [33], which are mathematically well defined [34,35], is usually fully sufficient. Gaussian processes can also be argued to be a natural starting point for reasoning on fields with known finite first and second order moments, as we will discuss now.

To be specific, let φ:Ω→R be a scalar field over some domain $\Omega \subset R^{u}$ and our prior knowledge on φ be the first and second moments of the field, e.g.,

(1) $$\begin{array}{ccc} {\langle \varphi^{x}\rangle }_{\left(\varphi \right)} & = & {\bar{\varphi }}^{x}\;\mathrm{and}\; \end{array}$$

(2) $$\begin{array}{ccc} {\langle {\left(\varphi -\bar{\varphi }\right)}^{x}{\left(\varphi -\bar{\varphi }\right)}^{y}\rangle }_{\left(\varphi \right)} & = & \Phi^{xy}\;\mathrm{for}\;\mathrm{all}\;x,y\in \Omega , \end{array}$$

with $\varphi^{x}:=\varphi \left(x\right)$ denoting a field value and ${\langle f(\varphi )\rangle }_{\left(\varphi \right)}:=\int D\varphi \;P\left(\varphi \right)f\left(\varphi \right)$ a prior expectation value for some function *f* of the field. If only the first and second field moments are given as prior information, it follows from the maximum entropy principle that the least informative probability distribution function encoding this information is a Gaussian with these moments. Thus, using this Gaussian

(3) $$\begin{array}{ccc} P(\varphi |I) & \equiv & G(\varphi -\bar{\varphi },\Phi )\\ & := & \frac{1}{\sqrt{\left|2\pi \Phi \right|}}\exp\left(-\frac{1}{2}{\left(\varphi -\bar{\varphi }\right)}^{†}\Phi^{-1}\left(\varphi -\bar{\varphi }\right)\right) \end{array}$$

as a prior with background information $I=({\langle \varphi \rangle }_{\left(\varphi \right)}=\bar{\varphi },{\langle \left(\varphi -\bar{\varphi }\right){\left(\varphi -\bar{\varphi }\right)}^{†}\rangle }_{\left(\varphi \right)}=\Phi )$ is a conservative choice, as it makes the least additional assumptions about the field except for the moments specified in *I*.

In many applications, however, the field of interest, the signal *s*, is not a Gaussian field, but may be related to such via a non-linear transformation. For example, in astronomical applications of IFT, the sky brightness field *s* is the quantity of interest, which is strictly positive, and therefore cannot be a Gaussian field. However, the logarithm of a brightness can be positive and negative and may therefore be modeled as a Gaussian process. In such a case, one could assign, e.g., $s^{x}\left(\varphi \right)=s_{0}\exp\left(\varphi^{x}\right)$ as a model for a diffuse (spatially correlated) sky emission component, with $s_{0}$ a reference brightness, chosen such that for example ${\langle \varphi^{x}\rangle }_{\left(\varphi \right)}=0$ holds.

Having established a field prior, Bayesian reasoning on the field φ, and therefore on the signal of interest s=s(φ), based on some data *d* and its likelihood P(d|φ,I) is possible. The field posterior

(4) $$P\left(\varphi |d,I\right)=\frac{P(d|\varphi ,I)P(\varphi |I)}{P(d|I)}$$

is defined as well as the prior and permits us to answer questions about the field, like its most probable configuration $\varphi_{\mathrm{MAP}}={\mathrm{argmax}}_{\varphi }P\left(\varphi |d,I\right)$ (MAP = maximum a posteriori), its posterior mean $m={\langle \varphi \rangle }_{\left(\varphi |d,I\right)}$, or its posterior uncertainty dispersion $D={\langle \left(\varphi -m\right){\left(\varphi -m\right)}^{†}\rangle }_{\left(\varphi |d,I\right)}$. IFT exploits the formalism of quantum and statistical field theory to calculate such posterior expectation values [1,28,36,37,38]. These formal calculations, however, should not be the focus here. Instead, it should be the formulation of IFT inference problems in terms of generative models, as these can be interpreted as GNNs.

For this purpose, the likelihood is expressed in terms of a measurement equation

(5) $$\begin{array}{ccc} d & = & R(\varphi )+n,\;\mathrm{with} \end{array}$$

(6) $$\begin{array}{ccc} R(\varphi ) & := & {\langle d\rangle }_{\left(d|\varphi \right)}, \end{array}$$

(7) $$\begin{array}{ccc} n & := & d-R(\varphi ),\;\mathrm{and} \end{array}$$

(8) $$\begin{array}{ccc} P(d|\varphi ,I) & \equiv & P(n=d-R(\varphi )|\varphi ), \end{array}$$

which is always possible if the data can be embedded into a vector space and the data expectation value ${\langle d\rangle }_{\left(d|\varphi \right)}$ exists. Here and in the following, we omit the background information *I* in probabilities. This rewriting of the likelihood in terms of a mean instrument response $d^{\prime }=R\left(\varphi \right)$ to the field φ and a noise process P(n|φ), which summarizes the fluctuations around that mean $d^{\prime }$, allows us to regard the data as the result of a noisy generative process that maps field values φ and associated noise realizations *n* onto data *d* according to Equation (5).

In case the instrument response and noise processes are provided for the signal *s* instead of the Gaussian field φ as $R^{\prime }\left(s\right):={\langle d\rangle }_{\left(d|s\right)}$ and P(n|s), their respective pull backs $R\left(\varphi \right):={\langle d\rangle }_{\left(d|s(\varphi )\right)}=R^{\prime }\left(s\left(\varphi \right)\right)$ and P(n|φ):=P(n|s(φ)) provide the necessary response and noise statistics w.r.t. the field φ.

All this provides a generative model for the signal *s* and data *d* via φ↩G(φ,Φ), s=s(φ), n↩P(n|s), and $d=R^{\prime }\left(s\right)+n$, which should now be standardized. The standardization introduces a generic latent space that permits better comparison to GNNs used in AI and ML and simplifies the usage of variational inference methods discussed later on.

### 2.2. Prior Standardization

Standardization of a random variable φ refers to finding a mapping from a standard normal distributed random variable ξ↩G(ξ,𝟙) to φ that reproduces the statistics of P(φ). For a Gaussian field φ, this is just a mapping of the form

(9) $$\varphi \left(\xi \right):=\bar{\varphi }+\Phi^{\frac{1}{2}}\xi ,$$

where $\Phi^{\frac{1}{2}}$ refers to a square root of Φ, which always exists for a covariance matrix that is positive definite. For the large class of band diagonal and therefore translational invariant covariance matrices Φ, which are very relevant for applications as we argue below, the square root of Φ can be explicitly constructed.

### 2.3. Power Spectra

In many signal inference problems, no spatial location is singled out a priori, before the measurement. This means that the field covariance between two locations only depends on the distance between these positions, but not on their absolute positions. Thus, $\Phi^{xy}=C_{\varphi }\left(x-y\right)$. As a consequence of the Wiener–Khinchin theorem, such a translational invariant field covariance becomes diagonal in harmonic space,

(10) $${\tilde{\Phi }}^{kq}=F_{x}^{k}\Phi^{xy}{\left(F^{†}\right)}_{y}^{q}={\left(2\pi \right)}^{u}\delta \left(k-q\right)P_{\varphi }\left(k\right)={\hat{P_{\varphi }}}^{kq}.$$

Here and in the following, F denotes a harmonic transform (a *u*-dimensional Fourier transform $F_{x}^{k}=\exp\left(ik\cdot x\right)$ in case of an Euclidean space, as we assume in the following), † the adjoint (complex conjugate and transposed of a matrix or vector), $P_{\varphi }\left(k\right):=F_{x^{\prime }}^{k}C_{\varphi }^{x^{\prime }}$ is the so called power spectrum of φ, the Einstein convention for repeated indices is used, as in ${\tilde{\varphi }}^{k}:=F_{x}^{k}\varphi^{x}\equiv \int dx^{u}\exp\left(ik\cdot x\right)\;\varphi \left(x\right)$, and $\hat{\phi }=\mathrm{diag}\left(\phi \right)$ denotes a diagonal operator in the space of the field ϕ with the values of ϕ on the diagonal.

Thanks to this diagonal representation of the field covariance in harmonic space, an explicit standardization of the field is given via

(11) $$\begin{array}{ccc} \xi & ↩ & G(\xi ,𝟙), \end{array}$$

(12) $$\begin{array}{ccc} \varphi & = & \bar{\varphi }+F^{-1}A_{\varphi }\;\xi ,\;\mathrm{and}\; \end{array}$$

(13) $$\begin{array}{ccc} A_{\varphi } & = & \hat{P_{\varphi }^{1/2}}, \end{array}$$

where the latter is an amplitude operator that is diagonal in harmonic space and that imprints the right amplitudes onto the Fourier modes of φ. This can be seen via a direct calculation,

(14) $$\begin{array}{ccc} {\langle \left(\varphi \left(\xi \right)-\bar{\varphi }\right){\left(\varphi \left(\xi \right)-\bar{\varphi }\right)}^{†}\rangle }_{\left(\xi \right)} & = & F^{-1}A_{\varphi }\;{\langle \xi \xi \rangle }_{\left(\xi \right)}A_{\varphi }^{†}F^{-1†}\;\\ \; & = & F^{-1}A_{\varphi }\;𝟙A_{\varphi }^{†}F^{-1†}\;\\ \; & = & F^{-1}\tilde{\Phi }F^{-1†}=\Phi \;\\ \; & = & {\langle \left(\varphi -\bar{\varphi }\right){\left(\varphi -\bar{\varphi }\right)}^{†}\rangle }_{\left(\varphi \right)}. \end{array}$$

In case no direction of the space is singled out a priori, the two-point correlation function and the power spectrum of φ become isotropic, $\Phi^{xy}=C_{\varphi }(|x-y|)$ and ${\tilde{\Phi }}^{kq}={\left(2\pi \right)}^{u}\delta \left(k-q\right)P_{\varphi }\left(|k|\right)$, respectively. In this case, only a one-dimensional power spectrum needs to be known. Such power spectra are often smooth functions on a double logarithmic scale in Fourier space, since any sharp feature in them would correspond to a (quasi-) periodic pattern in position space, which would be very unnatural for most signals. Thus, introducing the logarithmic Fourier space scale variable $\kappa \left(k\right):=\ln k/k_{0}$ w.r.t. some reference scale $k_{0}$, we expect

(15) $$\psi \left(\kappa \right):=\ln\left(P_{\varphi }\left(k_{0}e^{\kappa }\right)/P_{0}\right)$$

to be a field itself, in the sense that it is sufficiently smooth. Here, $P_{0}$ is a pivot scale for the power spectrum.

### 2.4. Amplitude Model

Often, the power spectrum as parameterized through ψ is not known a priori for a field φ, but statistical homogeneity, isotropy, and the absence of long range quasi-periodic signal variations make a Gaussian field prior for ψ plausible, $P\left(\psi \right)=G\left(\psi -\bar{\psi },\Psi \right)$. This log-log-power spectrum may exhibit fluctuations $\chi :=\psi -\bar{\psi }$ around a non-zero mean $\bar{\psi }\left(\kappa \right)$. The latter might, e.g., encode a preference for falling spectra and therefore for a spatially smooth field φ. In this case, just another layer for χ of a standardized generative model has to be added,

(16) $$\begin{array}{ccc} \eta & ↩ & G(\eta ,𝟙) \end{array}$$

(17) $$\begin{array}{ccc} \chi (\eta ) & := & A_{\psi }\eta ,\;\mathrm{with} \end{array}$$

(18) $$\begin{array}{ccc} A_{\psi }A_{\psi }^{†} & := & \Psi ,\;\mathrm{and} \end{array}$$

(19) $$\begin{array}{ccc} \psi (\eta ) & := & \bar{\psi }+\chi \left(\eta \right). \end{array}$$

Again, a prior for a field, here the only one dimensional χ(κ), is needed. A detailed description of how this amplitude model can be provided efficiently is given by [15]. This reference also provides a generative model for the case that the signal domain Ω is a product of sub-spaces, like position space and an energy spectrum coordinate, each requiring a different correlation structure, and the total correlation being a direct product of those. Assuming a direct product for the correlation structures might be possible for many field inference problems [15,39].

### 2.5. Dynamical Systems

Let us take a brief detour to fields shaped by dynamical systems. Dynamical systems, typically exhibit correlation structures that are not direct products of the spatial and temporal sub-spaces, as was proposed above. Here, the full spatial and temporal Fourier power spectrum $P_{\varphi }\left(k,\omega \right)$, with ω being the temporal frequency, encodes the full dynamics of a linear, homogeneous, and autonomous system. For example, a stochastic wave field φ(x,t) may follow the dynamical equation

(20) $$\left(\frac{\partial^{2}}{\partial t^{2}}+\eta \frac{\partial }{\partial t}-c^{2}\frac{\partial^{2}}{\partial x^{2}}\right)\varphi \left(x,t\right)=\xi \left(x,t\right),$$

where *c* is the wave velocity and η a damping constant. The field dynamics are determined by a response operator (or Green’s function) *G* that is a convolution of the exciting noise field ξ with a kernel *g*,

(21) φ=Gξ=g∗ξ,

where ∗ denotes convolution. In Fourier-space, this kernel can be applied by a direct point wise multiplication, ${\left(F\varphi \right)}^{\left(k,\omega \right)}={\left(Fg\right)}^{\left(k,\omega \right)}\;{\left(F\xi \right)}^{\left(k,\omega \right)}$ and is given by

(22) $$\begin{array}{ccc} {\left(Fg\right)}^{\left(k,\omega \right)} & = & {\left(\omega {}^{2}-i\eta \omega -c^{2}k^{2}\right)}^{-1}=:P_{G}\left(k,\omega \right). \end{array}$$

If the excitation of field fluctuations is caused by a white, stochastic noise field ξ↩G(ξ,𝟙), the resulting field has a power spectrum of

(23) $$P_{\varphi }\left(k,\omega \right)={\left|P_{G}\left(k,\omega \right)\right|}^{2}P_{\xi }\left(k,\omega \right)=\frac{1}{{\left(\omega {}^{2}-c^{2}k^{2}\right)}^{2}+\eta^{2}\omega^{2}}.$$

In this case, the spectrum is an analytical function in ω and *k*. This results from Equation (20) being a linear, homogeneous, and autonomous partial differential equation.

Linear integro-differential equations, however, can still be solved by convolutions, in which case the kernel might not have an analytically closed form any more, if the equation is still homogeneous and autonomous. For example, in neural field theory [40,41,42,43], a macroscopic description of the brain cortex dynamics, the neural activity φ(x,t) might be described by

(24) $$\frac{\partial }{\partial t}\varphi =-\varphi +w*\left(f\circ \varphi \right)+\xi .$$

Here, *w* is a spatial–temporal convolution kernel (that usually contains a delta function in time), f:R→R an activation function that is applied point wise to the field, (f∘φ)(x,t)=f(φ(x,t)), and we added an input term ξ. In case *f* is linear, the system responds linearly to inputs. Then, the input response is a convolution with a kernel *g* that has in general a non-analytical spectrum,

(25) $${\left(Fg\right)}^{\left(k,\omega \right)}={\left(1+i\omega -{\left(Fw\right)}^{\left(k,\omega \right)}\;f^{\prime }\right)}^{-1},$$

where $f^{\prime }$ is the slope of *f* and Fw the Fourier transformed kernel of the dynamics.

An inference of such non-analytical and highly structured response spectra from data is possible with IFT and can be used to learn the system dynamics from noisy system measurements [26,44]. It just requires a more complex spectral prior than discussed here. Let us now return to our main line of argumentation.

### 2.6. Generative Model

To summarize, the field inference problems of IFT can often be stated in terms of a standardized, generative model for the signal and the data. For the illustrative case outlined above, where the probabilistic model is given by

(26) $$\begin{array}{ccc} P(d,\varphi ,\psi ) & = & P(d|\varphi )P(\varphi |\psi )P(\psi ), \end{array}$$

(27) $$\begin{array}{ccc} P(\psi ) & = & G(\psi -\bar{\psi },\Psi ), \end{array}$$

(28) $$\begin{array}{ccc} P(\varphi |\psi ) & = & G(\varphi ,\Phi (\psi )), \end{array}$$

(29) $$\begin{array}{ccc} \Phi (\psi ) & = & F^{-1}\hat{P_{\varphi }}F^{-1†}, \end{array}$$

(30) $$\begin{array}{ccc} P_{\varphi }\left(k\right) & = & P_{0}\exp(\psi (\ln(|k|/k_{0}))),\;\mathrm{and} \end{array}$$

(31) $$\begin{array}{ccc} P(d|\varphi ) & = & P(d|s(\varphi )), \end{array}$$

the corresponding standardized generative model is

(32) $$\begin{array}{ccc} \zeta & := & (\xi ,\eta )↩G(\zeta ,𝟙), \end{array}$$

(33) $$\begin{array}{ccc} \psi (\eta ) & := & \bar{\psi }+A_{\psi }\eta , \end{array}$$

(34) $$\begin{array}{ccc} P_{\varphi }\left(k\right) & := & P_{0}\exp(\psi (\ln(|k|/k_{0})),\mathrm{is} \end{array}$$

(35) $$\begin{array}{ccc} \varphi (\xi ,\psi ) & := & F^{-1}\hat{P_{\varphi }^{1/2}}\xi , \end{array}$$

(36) $$\begin{array}{ccc} s(\varphi ) & := & s_{0}\exp\left(\varphi \right), \end{array}$$

(37) $$\begin{array}{ccc} n & ↩ & P(n|s),\;\mathrm{and} \end{array}$$

(38) $$\begin{array}{ccc} d & = & R^{\prime }\left(s\right)+n. \end{array}$$

**Figure 1:**
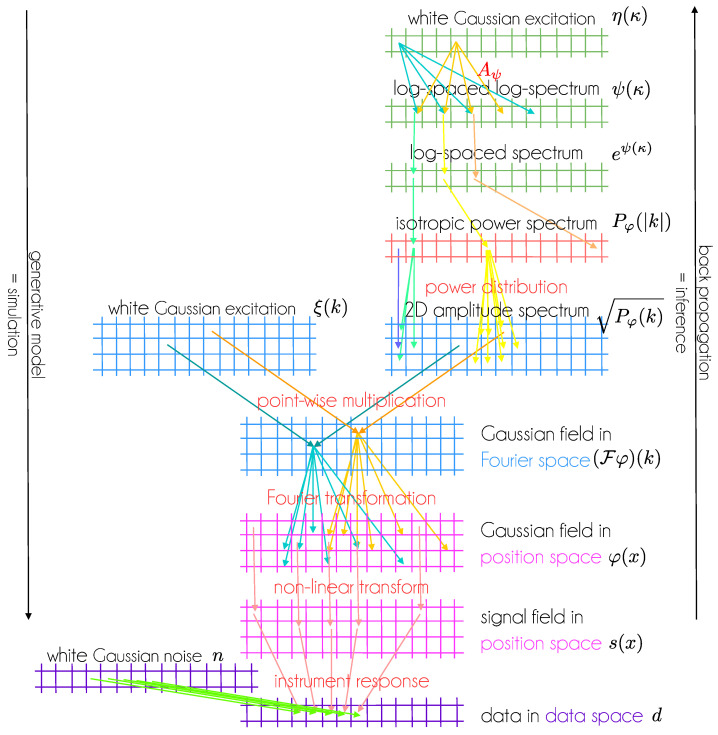
An IFT model for a 2D Gaussian random field also with generated homogeneous and isotropic correlation structure and its measurement according to Equations (32)–(38) displayed as a GNN. Layers with identical shapes are given identical colors. Note that all layers have a physical interpretation and the architecture of this GNN encodes expert knowledge on the field. Inserting random numbers into the latent spaces and executing the network from top to bottom corresponds to a simulation of signal and data generation. “Learning” the latent space variables from bottom to top via back propagation of data space residuals with respect to observed data corresponds to inference.

**Figure 2:**
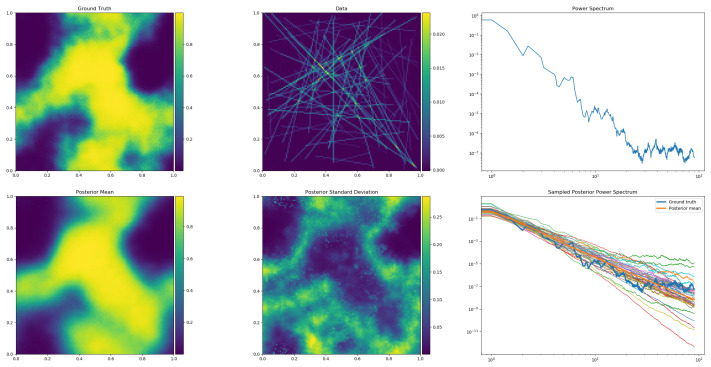
Output of a generative IFT model for a 2D tomography problem in simulation (**top row**) and reconstruction (**bottom rows**) mode. The model is depicted in Figure 1 and described by Equations (32)–(38) with the modification that in Equation (36) the exp-function is replaced by a sigmoid function to obtain more cloud-like structures. Run in simulation mode, the model first generates a non-parametric power spectrum (**top right panel**) from which a Gaussian realization of a statistical isotropic and homogeneous field is drawn (**top left**, after procession by the sigmoid function). This is then observed tomographically (**top middle**), by measurements that integrate over (here randomly chosen) lines of sight. The data values include Gaussian noise and are displayed at the locations of their measurement lines. Fed with this synthetic data set, the model run in inference mode (via geoVI) reconstructs the larger scales of the signal field (**bottom left**), the initial power spectrum (thick orange line in middle right panel; thick blue line is ground truth), and provides uncertainty information on both quantities (signal uncertainty is given at bottom middle, the power spectrum uncertainty is visualized by the set of thin lines at **bottom right**). The presented plots are the direct output of the getting_started_3.py script enclosed in the Numerical Information Field Theory (NIFTy) open source software package NIFTy8, downloadable at https://gitlab.mpcdf.mpg.de/ift/nifty (accessed on 17 December 2021) [46,47,48] that supports the implementation and inference of IFT models.

**Figure 3:**
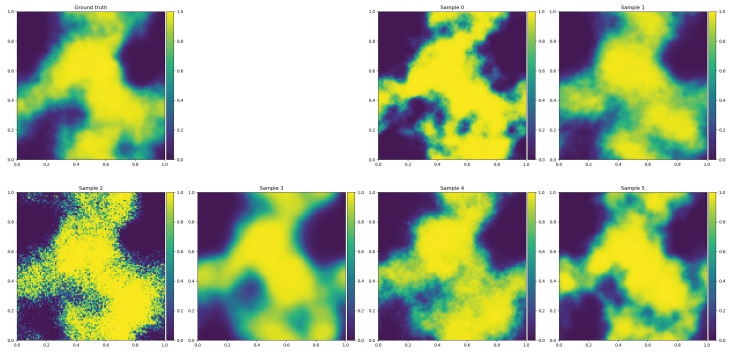
Signal ground truth (**top left panel**) and some signal posterior samples (**other panels**) of the field reconstructed in Figure 2. Note the varying granularity of the field samples due to the remaining posterior uncertainty of the power spectrum on small spatial scales as shown in Figure 2 at bottom right.

This generative model is illustrated in Figure 1. Variants of it are used in a number of real world data applications [5,6,7,8,9,10,11,12,13,14,15,16,17,18,19,20,21]. Its performance in generative and reconstruction mode is illustrated for synthetic data in Figure 2 and Figure 3.

For the noiseless data $d^{\prime }=R^{\prime }\left(s\right)$ the generative model reads

(39) $$\begin{array}{ccc} d^{\prime }\left(\zeta \right) & := & R^{\prime }\left(s\left(\varphi \left(\xi ,\psi \left(\eta \right)\right)\right)\right)\\ & = & \left(R^{\prime }\circ s\circ \varphi \circ f\right)\left(\zeta \right),\;\mathrm{with} \end{array}$$

(40) $$\begin{array}{ccc} f(\zeta ) & := & (\xi ,\psi (\eta )). \end{array}$$

This way, the full model complexity as given by Equations (26)–(31) is transferred into an effective response function $d^{\prime }=R^{\prime }\circ s\circ \varphi \circ f$. For this latent variable vector, the prior is simply P(ζ)=G(ζ,𝟙), whereas the likelihood $P\left(d|\zeta \right)=P(n=d-d^{\prime }\left(\zeta \right)|\zeta )$ has absorbed the full model complexity. This so called reparametrization trick [45] was introduced to IFT by [29] to simplify numerical variational inference.

At this point, it is essential to realize that this generative model consists of a latent space white noise process P(ζ)=G(ζ,𝟙) that generates an input vector ζ and a sequence of non-local linear and local non-linear operations that is applied to it. The Fourier transform $F^{-1}$ and $A_{\psi }$ are examples of non-local linear operations within the model. Among the non-linear operations are the exponential functions and the application of the ψ-dependent amplitude operator $A_{\varphi }\left(\psi \right)$ to the latent space excitations ξ, as there the two components of ζ=(ξ,η) are multiplied together. Furthermore, the instrument response $R^{\prime }\left(s\right)$ might also be decomposed into sequences of non-local linear and local non-linear operations, as physical processes in measurement devices can often be cast into the propagation of a quantity (an operation that is linear in the quantity) and the local interactions of the quantity (an operation non-linear in it), respectively.

## 3. Artificial Intelligence

### 3.1. Neural Networks

AI and ML are vast fields. AI aims at building artificial cognitive systems that perceive their environment, reason about its state and the systems’ best actions, and learn to improve their performance. ML can be regarded as a sub-field of AI, embracing many different methods like self-organized maps, Gaussian mixture models, deep neural networks, and many others. Here, the focus should be on specific neural networks, GNNs, as those have a close relation to the generative IFT models introduced before.

GNNs transform a latent space variable ξ↩G(ξ,𝟙) into a signal or data realization, s=s(ξ) or $d^{\prime }=d^{\prime }\left(\xi \right)$. A neural network is a function g(ξ) that can be decomposed in terms of *n* layer processing functions $g_{i}$ with

(41) $$g=g_{n}\circ g_{n-1}\circ \ldots g_{1}.$$

Any of the layer processing functions $g_{i}:\xi_{i}\mapsto \xi_{i+1}$ with $\xi_{1}\equiv \xi$ consists typically of a non-local, affine linear transformation $l_{i}\left(\xi_{i}\right):=L_{i}\xi_{i}+b_{i}$ of the input vector $\xi_{i}$ of layer *i* followed by a local, point wise application of non-linear, so-called activation functions $\sigma_{i}:R\to R$. Thus, the output vector $\xi_{i+1}$ of layer *i* is

(42) $$\begin{array}{ccc} \xi_{i+1} & = & \left(\sigma_{i}\circ l_{i}\right)\left(\xi_{i}\right), \end{array}$$

where $\sigma_{i}$ acts component wise. The set $\eta ={\left(L_{i},b_{i}\right)}_{i=1}^{n}$ of all coefficients of the $l_{i}$s (the matrix elements of the $L_{i}$ matrices, and the components of the $b_{i}$ vectors) determines the function the network represents. Putting the input values and network coefficients into a single vector ζ:=(ξ,η) a GNN can be regarded as a function of both, latent variables ξ and network parameters η, $d^{\prime }\left(\zeta \right)=g\left(\xi ;\eta \right)$.

### 3.2. Comparison with IFT Models

From this abstract perspective, a standardized, generative model $d^{\prime }\left(\zeta \right)$ in IFT is structurally a GNN, as both consist of sequences of local non-linear and non-local linear operations on their input vector ζ=(ξ,η). The concrete architecture of an IFT model and a typical GNN might differ significantly, as GNNs often map a lower dimensional latent space into a higher dimensional data or feature space, whereas the dimension of the IFT model latent space can be very high, as it contains a subset of the virtually infinite many degrees of freedom of a field, see Figure 1.

Additionally, the way IFT-based models and GNNs are usually used differs a bit. Both can be used to generate synthetic samples of outputs by processing random latent space vectors ξ↩G(ξ,𝟙). However, typically an IFT model $d^{\prime }\left(\zeta \right)$ is applied to infer all latent space variables in ζ from data *d*. From the latent variables, the signal of interest can always be recovered via s(ζ).

For this inference the so-called information Hamiltonian, potential, or energy

(43) $$\begin{array}{ccc} H(d,\zeta ) & = & -\ln P(d,\zeta )=-\ln P(d|\zeta )-\ln P(\zeta )\;\\ \; & = & H\left(n=d-d^{\prime }\left(\zeta \right)|\zeta \right)+\frac{1}{2}\zeta^{†}\zeta +\mathrm{const} \end{array}$$

is investigated with respect to ζ, where H(a|b):=−lnP(a|b). This quantity is introduced to IFT in analogy to statistical mechanics, it summarizes the full knowledge on the problem (as it is just a logarithmic coordinate transformation in the space of probabilities) and has the nice property, that it allows to speak about information as an additive quantity, as H(a,b)=H(a|b)+H(b).

Investigating the relevant information Hamiltonian for our IFT problem H(d,ζ) can be done, for example, by minimizing it to obtain a MAP estimator for ζ or—as discussed in the next section—via variational inference (VI). In case of a constant, signal independent Gaussian white noise statistics, the information Hamiltonian becomes

(44) $$\begin{array}{ccc} H(d,\zeta ) & = & \frac{|d-d^{\prime }{\left(\zeta \right)|}^{2}}{2\sigma_{n}^{2}}+\frac{1}{2}\zeta^{†}\zeta +\mathrm{const}. \end{array}$$

The training of an usual GNN is done with a training data set $\tilde{d}={\left(d_{i}\right)}_{i}$ to which a corresponding latent space vector set $\tilde{\zeta }={\left(\zeta_{i}\right)}_{i}$ and common network parameters η need to be found. For this a loss function of the form

(45) $$\begin{array}{ccc} \tilde{H}\left(\tilde{d},\tilde{\xi },\eta \right) & = & \sum_{i}\tilde{H}\left(d_{i}|\xi_{i},\eta \right)+\tilde{H}\left(\tilde{\xi }|\eta \right)+\tilde{H}\left(\eta \right), \end{array}$$

(46) $$\begin{array}{ccc} \tilde{H}\left(\tilde{\xi }|\eta \right) & = & \frac{1}{2}\sum_{i}\xi_{i}^{†}\xi +\mathrm{const},\;\mathrm{and} \end{array}$$

(47) $$\begin{array}{ccc} \tilde{H}\left(d_{i}|\xi_{i},\eta \right) & = & \frac{1}{2}\frac{|d_{i}-d^{\prime }\left(\xi_{i},\eta \right){|}^{2}}{2\sigma_{n}^{2}}+\mathrm{const} \end{array}$$

might be minimized. Here, a typical GNN data loss function $\tilde{H}\left({\tilde{d}}_{i}|\xi_{i},\eta \right)$ as used for the decoder part of an autoencoder (AE) [49] was assumed. In an generative adversarial network (GAN) [50], however, this data loss function is given in terms of the output of a discriminator network. The network parameter prior term $\tilde{H}\left(\eta \right)$ might be chosen to be uninformative ($\tilde{H}\left(\eta \right)=\mathrm{const}$) or informative (e.g., $\tilde{H}\left(\eta \right)=\frac{1}{2}\eta^{†}\eta$ in case of a Gaussian prior on the parameters).

Anyhow, by comparison of Equations (45)–(47) with Equations (43) and (44), it should be apparent that the network loss functions can be structurally similar to the IFT information Hamiltonian. Both consist of a standardized quadratic prior-energy and a likelihood-energy and both can have a probabilistic interpretation in terms of being negative log-probabilities, e.g.,

(48) $$\begin{array}{ccc} P(d,\xi ,\eta ) & = & e^{-H(d,\xi ,\eta )}\;\mathrm{and}\; \end{array}$$

(49) $$\begin{array}{ccc} P(\tilde{d},\tilde{\xi },\eta ) & = & e^{-\tilde{H}\left(\tilde{d},\tilde{\xi },\eta \right)}, \end{array}$$

respectively. For this reason, we do not distinguish between an information Hamiltonian H and a network loss function $\tilde{H}$ by writing H for both in the following.

The IFT-GNN can operate with solely a single data vector *d* due to the domain knowledge coded into their architecture, whereas usual GNNs require sets of data vectors $\tilde{d}={\left(d_{i}\right)}_{i}$ to be trained. Recently, more IFT-like architectures for GNNs were proposed as well, which are also able to process data without training [51].

## 4. Variational Inference

### 4.1. Basic Idea

So far, it has been assumed here that MAP estimators are used to determine network parameters ζ for both, IFT-based models as well as traditional GNNs. MAP estimators are known to be prone to over-fitting the data, as they are not probing the adjacent phase-space volumes of their solutions. VI methods perform better in that respect, while still being affordable in terms of computational costs for the high dimensional settings of IFT-based field inference and traditional GNN training. They were used in most recent IFT applications [12,13,14,15,16,17,18,21,52] and are prominently present in the name of variational autoencoders (VAEs) [45] that are built on VI.

In VI, the posterior P(ζ|d) is approximated by a simpler probability distribution $Q(\zeta |d^{\prime })$, in many applications by a Gaussian

(50) Q(ζ|θ,Θ)=G(ζ−θ,Θ),

where $d^{\prime }=\left(\theta ,\Theta \right)$. The Gaussian is chosen to minimize the variational Kullback–Leibler (KL) divergence

(51) $$\begin{array}{ccc} {\mathrm{KL}}_{\zeta }\left(d^{\prime },d\right) & := & D_{\mathrm{KL}}\left(Q||P\right)\;\\ \; & = & \int D\zeta \;Q\left(\zeta |d^{\prime }\right)\ln\frac{Q(\zeta |d^{\prime })}{P(\zeta |d)} \end{array}$$

with respect to the parameters of $d^{\prime }$, θ and Θ in our case.

Ideally, all degrees of freedom (DoF) of θ and Θ are optimized. In practice, however, this is often not feasible due to the quadratic scaling of the number of DoF of Θ with that of θ. Three approximate schemes for handling the high dimensional uncertainty covariance will be discussed in the following, leading to the ADVI, MGVI, and geoVI techniques introduced below, namely

- mean field theory, in which Θ is assumed to be diagonal, as used by ADVI
- the usage of the Fisher information to approximate Θ as a function of θ and thereby effectively removing the DoF of Θ from the optimization problem as used by MGVI
- a coordinate transformation of the latent space that approximately standardizes the posterior and therefore sets the covariance to the identity matrix in the new coordinates, as performed by geoVI.

Before these are discussed, a note that applies to all of them is in order. Optimizing of the VI KL, Equation (51), is slightly sub-optimal from an information theoretical point of view as this minimizes the amount of information introduced by going from P to Q. The expectation propagation (EP) KL with reversed arguments $D_{\mathrm{KL}}\left(P||Q\right)$ would be better, as it minimizes the information loss from approximating P with Q [53]. VI is known to underestimate the uncertainties, whereas EP conservatively overestimates them. However, calculating the EP solution for θ and Θ would require integrating over the posterior. If this would be feasible, any posterior quantity of interest could be calculated as well and there would be no need to approximate P(ζ|d) in the first place. Estimating and minimizing the VI KL $D_{\mathrm{KL}}\left(Q||P\right)$ is less demanding, as the integral over the simpler (Gaussian) distribution Q can very often be performed analytically, or by sample averaging using samples drawn from Q.

### 4.2. ADVI and Mean Field Approximation

In all here discussed VI techniques, the posterior mean θ and the posterior uncertainty covariance Θ become parameters to be determined. The vector θ has the dimension $N_{\dim}$ of the latent space, whereas the posterior uncertainty covariance Θ has $N_{\dim}\left(N_{\dim}-1\right)/2=O\left(N_{\dim}^{2}\right)$ independent DoF. For small problems, these might be solved for, however, for large problems with millions of DoF, these cannot even be stored in a computer memory. To circumvent this, the Automatic Differentiation Variational Inference (ADVI) algorithm [54] often invokes the so called mean field approximation (MFA). This assumes a diagonal covariance $\Theta_{\mathrm{MFA}}=\hat{\theta^{\prime }}\equiv \mathrm{diag}\left(\theta^{\prime }\right)$, with $\theta^{\prime }$ being a latent space vector. Cross-correlations between parameters can not be represented by this, which is problematic in particular in combination with the tendency of VI to underestimate uncertainties.

### 4.3. MGVI and Fisher Information Metric

In order to overcome this limitation of ADVI that limits its usage in IFT contexts with their large number of DoF, the Metric Gaussian Variational Inference (MGVI) [30] algorithm approximates the posterior uncertainty of ζ with the help of the Fisher information metric

(52) $$\begin{array}{ccc} M(\zeta ) & := & {\langle \frac{\partial H(d|\zeta )}{\partial \zeta }{\frac{\partial H(d|\zeta )}{\partial \zeta }}^{†}\rangle }_{\left(d|\zeta \right)}. \end{array}$$

The starting point for obtaining the uncertainty covariance Θ used in MGVI is the Hessian of the log-posterior

(53) $$\begin{array}{ccc} \frac{\partial^{2}H\left(\zeta |d\right)}{\partial \zeta \partial \zeta^{†}} & = & \frac{\partial^{2}H\left(d,\zeta \right)}{\partial \zeta \partial \zeta^{†}}-\underset{=0}{\underset{︸}{\frac{\partial^{2}H\left(d\right)}{\partial \zeta \partial \zeta^{†}}}}\\ & = & \frac{\partial^{2}H\left(d,\zeta \right)}{\partial \zeta \partial \zeta^{†}} \end{array}$$

as a first guess for the approximate posterior precision matrix $\Theta^{-1}$. Using this evaluated at the minimum $\zeta_{\mathrm{MAP}}$ of the information Hamiltonian H(d,ζ) would correspond to the Laplace approximation, in which the posterior is replaced by a Gaussian obtained from doing a saddle point approximation at its maximum.

However, neither is the MAP solution ideal, as discussed above, nor would this be a good approximation at many locations ζ that differ from $\zeta_{\mathrm{MAP}}$. This is because positive definiteness of the Hessian is not guaranteed there, but it is an essential property of any correlation and precision matrix. For this reason, $\Theta^{-1}$ cannot directly be approximated by this Hessian.

It turns out that the likelihood averaged Hessian is strictly positive definite, and is therefore a candidate for an approximate posterior precision matrix for any guessed posterior mean θ. A short calculation shows that the likelihood averaged Hessian is indeed positive definite:

(54) Θ−1(θ)≈∂2H(d,ζ)∂ζ∂ζ†(d|ζ=θ)=∂2H(ζ)∂ζ∂ζ†+∂2H(d|ζ)∂ζ∂ζ†(d|ζ=θ)=𝟙−∂2lnP(d|ζ)∂ζ∂ζ†(d|ζ=θ)=𝟙−1P(d|ζ)∂2P(d|ζ)∂ζ∂ζ†(d|ζ=θ)+1P2(d|ζ)∂P(d|ζ)∂ζ∂P(d|ζ)∂ζ†(d|ζ=θ)=𝟙−∫ddP(d|ζ)P(d|ζ)∂2P(d|ζ)∂ζ∂ζ†+∂H(d|ζ)∂ζ∂H(d|ζ)∂ζ†(d|ζ=θ)=𝟙−∂2∂ζ∂ζ†∫ddP(d|ζ)︸=1︸=0+M(θ)=𝟙+M(θ)>0.

The last step follows because the Fisher metric M(θ) is an average over outer products ($v\;v^{†}\geq 0$) of likelihood Hamiltonian gradient vectors v=∂H(d|ζ)/∂ζ and thereby positive semi-definite. Adding 𝟙>0 to the Fisher metric turns the approximate precision matrix into a positive definite matrix $\Theta^{-1}\left(\theta \right)>0$, of which the inverse Θ(θ) exists for all θ, and which is positive definite as well.

### 4.4. Exact Uncertainty Covariance

Being positive definite is of course not the only property an approximation of the posterior uncertainty covariance has to fulfill. It also has to approximate well. Fortunately, this seems to be the case in many situations. The likelihood averaged Laplace approximation actually becomes the exact posterior uncertainty in case of linear Gaussian measurement problems as is shown in the following. If it is exact in such linear situations, it should be a valid approximation in the vicinity of any linear case.

For linear measurement problems, the measurement equation is of the form d=Rζ+n, the noise statistics P(n|ζ)=G(n,N), and the standardized prior is P(ζ)=G(ζ,𝟙). The corresponding posterior is known to be a Gaussian

(55) P(ζ|d)=G(ζ−m,D)

with mean *m* and covariance *D* given by the generalized Wiener filter solution $m=D\;R^{†}N^{-1}d$ and the Wiener covariance $D={\left(𝟙+R^{†}N^{-1}R\right)}^{-1}$, respectively (e.g., [1]). In this case, the Fisher information metric $M=R^{†}N^{-1}R$ is independent of ζ. The approximate posterior uncertainty covariance as given by Equation (54) equals the exact posterior covariance, $\Theta ={\left(𝟙+M\right)}^{-1}={\left({𝟙}^{-1}+R^{†}N^{-1}R\right)}^{-1}=D$. Thus indeed, the adopted approximation becomes exact in this situation. This should show why this approximation can hold sensible results in sufficiently well behaved cases, in particular when a linearization of the inference problem around a reference solution (e.g., a MAP estimate) is already a good approximation.

Furthermore, for all signal space directions around this reference point that are unconstrained by the data, this covariance approximation returns the prior uncertainty, as it should. Additional discussion of this approximation can be found in Knollmüller and Enßlin [30], where also its performance with respect to ADVI is numerically investigated.

The important point about this approximate uncertainty covariance Θ is that it is a function of the latent space mean estimate θ, i.e., Θ(θ), and therefore does not need to be inferred as well. For many likelihoods, the Fisher metric is available analytically, alleviating the need to store Θ in a computer memory as an explicit matrix. It is only necessary that certain operations can be performed with Θ, like applying it to a vector or drawing samples from a Gaussian with this covariance. Relying solely on those memory inexpensive operations, the MGVI algorithm is able to minimize the relevant VI KL, namely ${\mathrm{KL}}_{\zeta }\left(\left(\theta ,\Theta \left(\theta \right)\right),d\right)=D_{\mathrm{KL}}\left(Q,P\right)$, with respect to the approximate posterior mean θ. The result of MGVI are then the posterior mean θ, the uncertainty covariance Θ(θ), and posterior samples ${\left\{\zeta_{i}\right\}}_{i}$ drawn according to this mean and covariance. These samples can then be propagated into posterior signal samples $s_{i}=s\left(\zeta_{i}\right)$, from which any desired posterior signal statistics can be calculated.

MGVI has enabled field inference for problems, which are too complex to be solved by MAP, in particular when multiple layers of hyperpriors were involved (e.g., [13,15]).

### 4.5. Geometric Variational Inference

ADVI’s and MGVI’s weak point, however, can be the Gaussian approximation of the posterior, which might be strongly non-Gaussian in certain applications. In order to overcome this, the geometrical variational inference (geoVI) algorithm [32] was introduced as an extension of MGVI. geoVI puts another coordinate transformation on top of the one used by MGVI, so that $\zeta =g_{0}\left(y\right)$—with $g_{0}$ to be performed before any of the other IFT-GNN operations $g_{1},\ldots g_{n}$ — approximately standardizes the posterior, P(y|d)≈G(y,𝟙). Astonishingly, this transformation can be constructed without the (prohibitive) usage of any explicit matrix or higher order tensor in the latent space, thus also allowing us to tackle very high dimensional inference problems, like MGVI. The transformation is basically a normalizing flow (network) [55], just with the difference to their usual usage in ML, that the geoVI flow does not need to be trained, but is derived from the problem statement in form of its information Hamiltonian in an automated fashion. Specifically, the coordinate transformation $g_{0}$ is defined to solve the constraining equation

(56) $${\left.{\frac{\partial g_{0}}{\partial y}}^{†}\Theta \left(\zeta \right)\frac{\partial g_{0}}{\partial y}\right|}_{\zeta =g_{0}\left(y\right)}\approx 𝟙\;\forall y,$$

which fully specifies $g_{0}$ up to an integration constant θ. This remaining constant is solved for by minimizing the VI KL with respect to θ to retrieve the optimal geoVI aproximation.

With geoVI, deeper hierarchical models, which more often exhibit non-Gaussian posteriors due to a larger number of degenerate parameters in them, can be approached via VI. The ability of geoVI to provide uncertainty information is illustrated in Figure 2 (bottom middle and right panels) and in Figure 3. Further details on geoVI and detailed comparisons of ADVI, MGVI, geoVI, and Hamiltonian Monte Carlo methods can be found in [32].

## 5. Conclusions and Outlook

This paper argues that IFT techniques can well be regarded as ML and AI methods by showing their interrelation with GNNs, normalizing flows, and VI techniques. This insight is not necessarily new, as this paper just summarizes a number of recent works [29,30,31,32] that suggested this before.

First, the generative models build and used in IFT are GNNs that can interpret data without initial training, thanks to the domain knowledge coded into their architecture [29]. Related architectures have very recently been proposed as image priors in the context of neural network architectures as well [51]. As IFT models and the newly proposed image priors do not obtain their intelligence from data driven learning, they are strictly not ML techniques, but might be characterized as (expert) knowledge-driven AI systems. From a technical point of view, however, such a distinction could be seen as splitting hairs.

Second, the VI algorithms used in IFT and AI to approximately infer quantities are a natural interface between these areas. Here, the related ADVI [54], MGVI [30], and geoVI [32] algorithms were briefly discussed, which can be used in classical ML and AI as well as in IFT applications.

And third, the common probabilistic formulation of IFT models and GNNs, as well as the common VI infrastructure of the two areas allows for combining pre-trained GNNs and other networks with IFT-style model components. In that respect, the possibility to perform Bayesian reasoning with trained neural networks as described in [31] might give an outlook on the potential to combine IFT with other ML and AI methods.

To summarize, IFT [1,2] addresses perception [5,6,7,8,9,10,11,12,13,14,15,16,17,18,19,20,21], reasoning [22,23,24,25,26,31], and adaptive inference [30,32] tasks. All these are central to the aims of AI and ML to build intelligent systems including such for perception, cognition, and learning.

## Data Availability

The presented data are synthetically generated via the script getting_started_3.py enclosed in the open source software package NIFTy in its version 8, downloadable at https://gitlab.mpcdf.mpg.de/ift/nifty/-/tree/NIFTy_8 (accessed on 17 December 2021).
